# Evaluating Large Language Models in the Image-Based Diagnosis of Intracranial Tumors

**DOI:** 10.7759/cureus.97449

**Published:** 2025-11-21

**Authors:** Cameron A Rivera, Vratko Himic, Nathan T Zwagerman, Ashish H Shah, Michael E Ivan, Ricardo J Komotar, Daniel M Aaronson

**Affiliations:** 1 Neurological Surgery, University of Miami Miller School of Medicine, Miami, USA; 2 Neurosurgery, Medical College of Wisconsin, Milwaukee, USA

**Keywords:** artificial intelligence (ai), chatgpt, image analysis, large language model (llm), neuroimaging, neuro-oncology

## Abstract

Background

Artificial intelligence (AI) tools exist at the intersection of machine learning and natural language processing and are poised to rapidly transform healthcare. There has been a growing interest from clinicians in the ability of patient-accessible image analysis tools embedded into large language models (LLMs) to interpret raw clinical neuroimaging. Here, we compare the performance of GPT-4V and GPT-4o to that of neurosurgical attendings and trainees.

Methodology

A total of 20 brain MRI scans were included in this analysis, consisting of five gliomas, five meningiomas, five pituitary tumors, and five non-tumor control images. GPT-4V and GPT-4o were provided with identical prompts and MRI scans to determine the most likely diagnosis. Model performance in classifying each MRI scan into one of these four categories was compared to survey responses from neurosurgery attendings, fellows, senior residents, and junior residents.

Results

GPT-4V correctly diagnosed 40% of cases (n = 20), whereas GPT-4o achieved a 70% accuracy rate (n = 20). Neurosurgery attendings, fellows, and residents (n = 14) collectively identified the correct diagnoses in 84.6% of cases across the same 20 images (n = 280) based on a single cross-sectional MRI scan. Mean Cohen’s kappa of surgeons compared to GPT-4V was 0.18, and compared to GPT-4o was 0.51.

Conclusions

While LLMs underperformed compared to surgeons in identifying central nervous system malignancies, GPT-4o demonstrated substantial improvement over GPT-4V, highlighting the rapid advancement of AI capabilities. Interrater reliability statistics showed further evidence that GPT-4o closely resembles human-level performance than GPT-4V. Further refinement of these models may bridge the performance gap and expand their utility in clinical neuroimaging. Extra caution should be given to patients in the use of such models at the individual patient level.

## Introduction

The rise of large language models (LLMs) such as GPT-4 has introduced many new tools to the field of neurosurgical oncology. Recent advancements in these commercially available artificial intelligence (AI) software solutions have expanded their capabilities to include image analysis, such as interpreting MRI scans, without the need for explicit supervised learning from a labeled database. Especially given the recently reported success of LLMs on written neurosurgical board examinations [1] and the public surge of interest in consumer-friendly AI technologies, surgeons are increasingly interested in understanding the technology available to their patients and the public at large. When accessed privately by patients, this technology can generate further questions that they may wish to discuss with their surgical team.

Numerous studies have explored the use of machine learning (ML) imaging models in radiology [2,3], oncology [4], and neurosurgery [5,6]. These studies often utilize convolutional neural networks to classify radiomics data; however, these models are often trained to solve one specific task and are trained with a limited quantity and variety of images. Generalizability to, and integration with, clinical practice is often cited as a limiting factor to radiomics ML models [7]. However, these models are not published publicly for interactive use and are not integrated into sophisticated chat interfaces that LLMs provide, despite evidence that a combined text and image-based ML approach can provide diagnostic value [8].

LLMs have spiked in popularity with the publication of ChatGPT, a widely popular text-based interface that allows users to input text and receive customized responses from the pretrained transformer model. Expansion of the interface in models such as GPT-4V allows users to upload image files, which are analyzed by the model, forming the model’s text response based on the uploaded image. While the text-based services of GPT-4V have succeeded in responding to radiology queries [9], GPT-4V has exhibited poor performance in successfully analyzing clinical image inputs [10-12]. Its successor, GPT-4o, has demonstrated promising improvements in various medical imaging studies [13,14] and is, at the time of publishing, available to the public at no cost.

With the increasing accessibility of AI technologies, individuals are now able to conveniently interact with AI tools to gain quick insights into their medical conditions before consulting a medical professional. Patients can upload medical images, such as cranial MRI scans, with no contextual medical information and receive immediate interpretations. While this widely available access to machine visual processing empowers patients, it remains unclear how accurate these models are in informing the public at an individual user level. Here, we have sought to explore and understand the current capabilities of LLMs that are available to the public and their use in neuro-oncologic image analysis, comparing their performance to that of residents, fellows, and attending neurosurgeons in a proof-of-concept study.

## Materials and methods

Image selection and neurosurgeon survey

**Figure 1:**
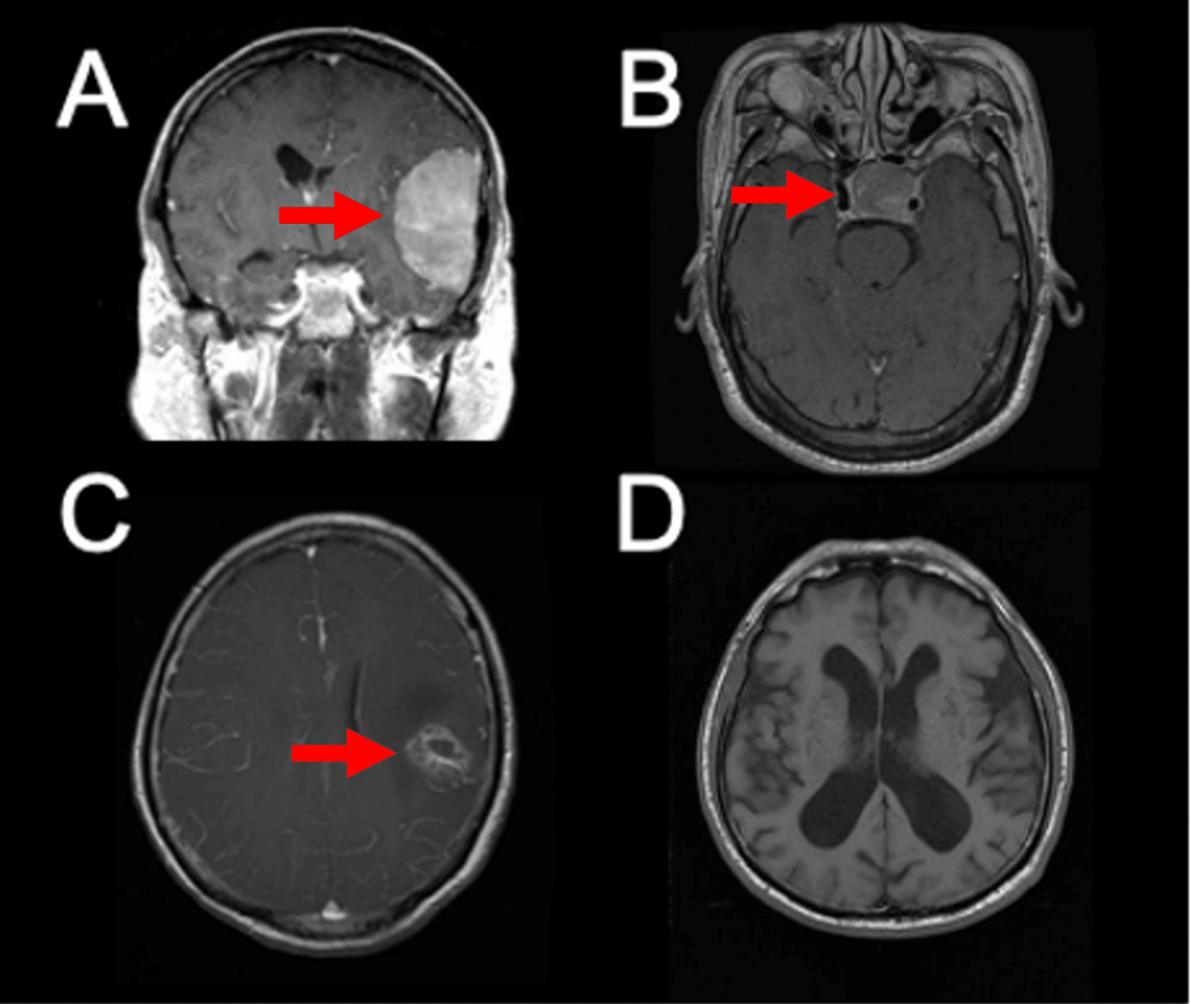
Sample MRI survey scans. Sample images provided to both neurosurgeons, trainees, and the large language models, including (A) meningioma, (B) pituitary tumor, (C) glioma, and (D) non-tumors. Intracranial tumors for panels A-C are indicated with a red arrow for illustration, but were not marked or annotated during the evaluation.

MRI scans were selected at random from the publicly available brain tumor MRI dataset [15]. A 20-question survey was compiled of single-slice MRI scans, either in the axial or coronal plane, comprising 15 tumor pathologies and five non-tumor controls. Images were not preprocessed or standardized before utilization in the study. Surgeons were asked to diagnose glioma, pituitary adenoma, meningioma, metastasis, or non-tumor for each of the images (Figure 1). Surgeons completed this survey in the spring of 2024. Survey data is available upon reasonable request to the senior author.

GPT-4V and GPT-4o query

Both GPT-4V [16] and GPT-4o [17] models were queried with a standardized prompt for all 20 images that were shown to the surgeon cohort: “For each of the following, please type out what you think the underlying most likely single diagnosis is.” This query took place in the spring of 2024. One image was provided for each case, selected at random from T1, contrast-enhanced T1, T2, or fluid-attenuated inversion recovery sequences. Images were not marked or annotated. Survey responses were recorded from the model’s text output.

Statistical analysis

Accuracy was calculated per respondent from survey responses and binned by surgical training level. Mean and standard deviation of aggregate surgeon responses were reported. For each model, Cohen’s kappa statistic was calculated between each surgeon and the model; results were averaged with the standard deviation reported.

## Results

Data collection

The MRI survey consisted of five gliomas, five pituitary adenomas, five meningiomas, and five non-tumor pathologies. No images of metastasis were included. In total, 14 neurosurgeons completed the imaging survey: five junior residents, two senior residents, four fellows, and three attending surgeons. Both GPT-4V and GPT-4o were successfully queried for survey responses.

Survey results

**Figure 2:**
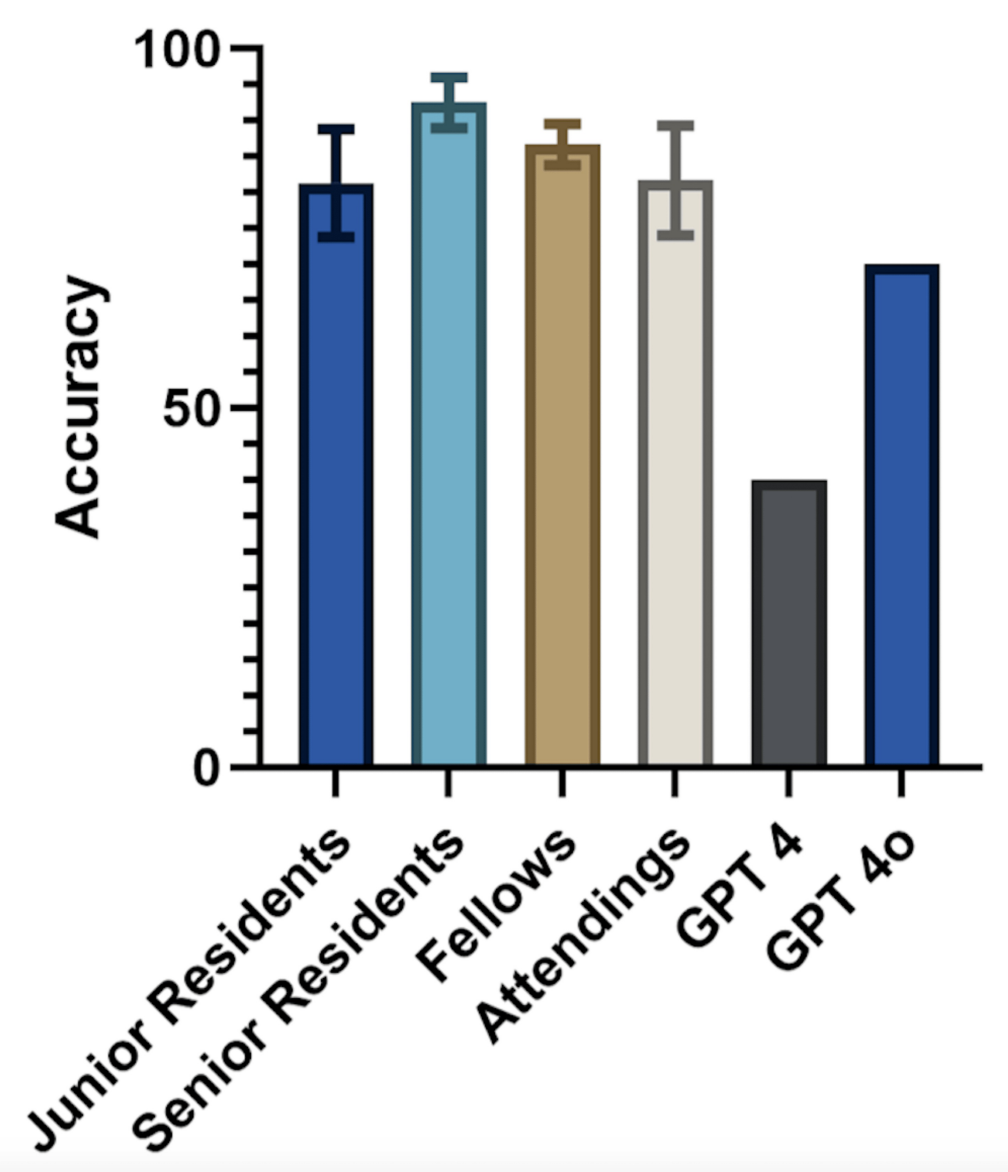
MRI scan analysis survey results. Mean accuracy of survey respondents stratified by training level compared to two large language models tested in this analysis. Standard deviation is plotted for surgeon responses.

Across the 20 test images, the mean surgeon accuracy in selecting the correct diagnoses across the images presented was 84.6% (standard deviation = 6.89%). The mean Cohen’s kappa statistic, which quantifies the congruence of how well two reporters agree when classifying items into categories, was 0.80 (standard deviation = 0.09). GPT-4V achieved an accuracy of 30% with a Cohen’s kappa statistic of 0.138, with GPT-4o scoring 70%, with a Cohen’s kappa statistic of 0.61. Stratification of surgeon accuracy by level of training is displayed in Figure 2. The average Cohen’s kappa score between GPT-4V and surgeons was 0.18 ± 0.07; the average for GPT-4o was 0.51 ± 0.07.

## Discussion

When comparing the performance of neuro-oncologic image analysis to surgeons of various experience levels in this proof-of-concept study, GPT-4o demonstrated accuracy close but inferior to that of trained neurosurgeons, while GPT-4V showed poor performance in tumor diagnosis. The inconsistency of GPT-4V in basic image analysis tasks has been previously documented, where it has been shown to respond confidently with diverging answers to identical yet differently worded prompts [15]; on the contrary, recent studies in other medical fields have reported a diagnostic accuracy of GPT-4o similar to the results found in our study [12,13]. The surgeons responding to our survey consistently showed superior diagnostic performance regardless of training level. While the accuracy of GPT-4o (70%) remained notably higher than previous models, surgeons should be made aware of the limitations of patient-accessible image diagnostic models.

An interesting finding of this analysis was the increased average Cohen’s kappa statistic for GPT-4o compared to GPT-4V. Given the role of Cohen’s kappa in evaluating the level of agreement between two raters, this evidence suggests a closer semblance of GPT-4o’s performance to a human, regardless of accuracy, compared to its predecessor, GPT-4V. This result suggests a possibly increasing semblance to human performance for many data-driven tasks with each GPT model iteration. As discussed elsewhere, these models have an infinite upward potential to continue improving with access to increasing quantities of data [13].

In discerning the differences between GPT-4V and GPT-4o, we must consider both the changes in model architecture and training data. GPT-4V adds a visual perceptron to the existing text capabilities of GPT-4, whereas GPT-4o is deemed an “autoregressive omni model, which accepts as inputs any combination of text, audio, image, and video” [17]. OpenAI has directly addressed the shortcomings of GPT-4V in medical imaging, publishing an example in their report of a mislabeled image of hydrocephalus on axial MRI [18]. Still, published work outside neurosurgery has shown GPT-4V’s ability to pass both basic and advanced life support exams (such as Basic Life Support and Advanced Cardiovascular Life Support) with a 90% accuracy, including image-based questions [19]. Despite keeping most details proprietary, OpenAI has released reports confirming that GPT-4o is trained by private industry data sharing agreements, including images from Shutterstock repositories [16]. Regarding text-based queries, OpenAI has even published the results of GPT-4o in answering United States Medical Licensing Examination questions, suggesting that GPT-4o developers have considered the healthcare utility of its product.

Looking ahead to the interplay between AI models and patients, we must consider the accuracy of the tools available to our patients. Recent literature has emphasized that AI tools can be appropriate for population-level insights but poorly suited for individual patient analysis [20]. Proper guidance must be communicated to the public about the inconsistency of large models at the individual patient level, as these models exist in their current form, particularly in the outpatient setting. Moreover, a recent meta-analysis and literature review has found that over 99% of deep learning radiology models have not been properly validated externally [21], suggesting that more rigorous experimentation is required to avoid misleading patients before their contact with medical professionals. Patients must be made aware of the potential risks of anchoring bias and the confusion that could arise from misinterpretations by these models.

Much progress has been made over the past few decades to establish a less patriarchal approach to medical care, where patients are encouraged and invited to understand, research, and engage with their conditions, with ultimate emphasis on patient autonomy. However, with expanded internet access to online tools such as WebMD or ChatGPT, unsupervised self-triage has the potential for misdiagnosis and patient harm [22,23]. This shift in at-home resources can lead to complex discussions between medical professionals and patients, especially if AI-generated insights conflict with clinical recommendations. Navigating these conversations tactfully will be an essential skill for healthcare providers in the near future. Strengthening collaboration between medical institutions and emerging technologies will be crucial in maintaining patient trust as these advancements continue to evolve.

This study is limited by our narrow understanding of the underlying LLM architecture and its training data, as well as by our limited sample size. Regarding each clinical course, the deidentified nature of the studied images prevents any correlated clinical context from being provided to either the AI models or surgeon participants. Additionally, AI models may be continuously updated or improved, potentially affecting the reproducibility of the recorded results.

## Conclusions

In our evaluation of pre-trained AI image analysis, we found that GPT-4V performed poorly at identifying and differentiating intracranial neoplasms, while the newer GPT-4o correctly identified pathologies. Interestingly, GPT-4o's performance more closely mirrored that of a surgeon compared to GPT-4V. While LLM-based image analysis tools still lag behind surgeons in recognizing and classifying tumor pathology on clinical MRI, these publicly available tools are advancing rapidly and becoming increasingly accessible. As these technologies evolve, patients and the public must exercise caution and be aware of their limitations, such as those highlighted in this study. Clinicians, particularly in neuro-oncology, must stay informed about these advancements to better understand the tools available to our patients. Engaging with patients at their level of understanding will be essential in guiding discussions, upholding patient autonomy, and optimizing personalized tumor management in neuro-oncology.
